# Active and Robust Composite Films Based on Gelatin and Gallic Acid Integrated with Microfibrillated Cellulose

**DOI:** 10.3390/foods10112831

**Published:** 2021-11-17

**Authors:** Yinghua Luo, Yanbei Wu, Yali Wang, Liangli (Lucy) Yu

**Affiliations:** 1 College of Food Science and Nutritional Engineering, National Engineering Research Centre for Fruits and Vegetables Processing, Key Laboratory of Storage and Processing of Fruits and Vegetables, Ministry of Agriculture, Engineering Research Centre for Fruits and Vegetables Processing, Ministry of Education, China Agricultural University, Beijing 100083, China; luoyinghua@cau.edu.cn; 2 School of Food and Health, Beijing Technology and Business University, Beijing 100048, China; wyali1125@163.com (Y.W.); 3 Department of Nutrition and Food Science, University of Maryland, College Park, MD 20742, USA; lyu5@umd.edu

**Keywords:** gelatin, gallic acid, active film, microfibrillated cellulose, reinforcement

## Abstract

Background: Gelatin is a renewable, biodegradable, and inexpensive food polymer. The insufficient mechanical and functional properties of gelatin-based films (GBF) restrict their commercial application in food packaging. This work proposed a facile strategy to prepare an active and robust GBF that has the potential to be used in food packaging. Methods: A strong and active GBF was prepared based on the principle of supramolecular chemistry via the incorporation of gallic acid (GA) as an active crosslinking agent and of microfibrillated cellulose (MFC) as a reinforcing agent. Results: Under the appropriate concentration (1.0 wt%), MFC was evenly dispersed in a gelatin matrix to endow the film with low surface roughness and compact structure. Compared with the GF, the tensile strength and elongation at break of the resultant film reached 6.09 MPa and 213.4%, respectively, representing the corresponding improvement of 12.8% and 27.6%. Besides, a significantly improved water vapor barrier (from 3.985 × 10^−8^ to 3.894 × 10^−8^ g·m^−1^·Pa^−1^·s^−1^) and antioxidant activity (from 54.6% to 86.4% for ABTS radical scavenging activity; from 6.0% to 89.1% for DPPH radical scavenging activity) of GBFs were also observed after introducing the aromatic structure of GA and nano-/microfibrils in MFC. Moreover, the UV blocking performance and thermal stability of GGF and GGCFs were also enhanced. Conclusions: this work paves a promising way toward facile preparation of multifunctional GBFs that have great potential to be used in fabricating active and safe food packaging materials for food preservation.

## 1. Introduction

In the food industry, petroleum-based plastic film has been the frontrunner for decades now due to its low price and high stability [1]. However, the huge consumption of plastic food packaging materials has caused serious “white pollution”, which leads to increasing threats to human health and the ecosystem [2]. Thus, it poses a pressing need for developing green packaging materials based on biodegradable and renewable biopolymers, e.g., polysaccharides and proteins. Gelatin, a film-forming biomaterial derived from animal meat production byproducts [3], has been widely used in preparing food packaging materials with good biodegradability in recent years due to its low cost, excellent film-forming properties, renewability, biocompatibility, high transparency, etc. [4,5].

However, gelatin has the inherent drawback of brittle nature (low strength and flexibility) owing to its low water resistance caused by its hydrophilicity [6,7]; thus, its wide application as a food packaging film material is limited. Given this, the modification of gelatin films (GFs) is essential for improving their application performance. Aldehydes [8,9,10], polyphenols [11,12], and polysaccharides [13,14] are the most widely used modification agents. Besides, various hydrophobic compounds such as fats, oils, and waxes have been incorporated into gelatin networks to improve their water barrier properties, thus enabling the full utilization of these networks for food packaging applications [15,16]. Among them, aldehydes are the most widely used modification reagents since aldehyde-containing groups can react with amino groups to form stable Schiff base covalent bonds [17,18], thus improving the crosslinking degree of gelatin molecules. Owing to this beneficial effect, the introduction of aldehydes at a low concentration can enhance the thermal stability and mechanical properties of GFs. Nowadays, food safety is attracting more and more serious attention; the safety of these highly active aldehyde reagents, especially of glutaraldehyde that features cytotoxicity, has been highly concerning [19]; modification reagents must be carefully selected when used in food packaging films. As for the modification of GF using wax, the modified GF had dissatisfactory mechanical strength [15]. Hence, fabricating gelatin-based films (GBFs) that are both safe and robust is still a research challenge.

In addition to covalent crosslinking, the formation of multipoint hydrogen bonds between the modification reagents and gelatin molecules can also endow the GBFs with improved mechanical properties and thermal stability. Polyphenol-containing abundant phenolic hydroxyl groups can play the aforementioned role. Besides, polyphenol has excellent and attractive health-related properties, e.g., antioxidant, antibacterial and antitumor activities [20]. In addition to generating multipoint hydrogen bonds with gelatin molecules to form a more stable spatial structure, polyphenol can endow the crosslinked film materials with considerable antioxidant activity to reduce the oxidative degradation of food during storage. Meanwhile, it also lowers the risk of antioxidants addition to food during production and improves the safety of packaging films. In view of this, grape seed extracts and pine needle extracts containing abundant polymeric tannins were incorporated into gelatin films to prepare active gelatin-based films for food packaging [21,22,23]. In general, a food packaging film must be robust enough to withstand possible mechanical damage during food processing, storage and distribution. However, it has been reported that it is not sufficient to meet the requirements of food packaging materials by only relying on the multipoint hydrogen bonds between polyphenols and gelatin to enhance the mechanical properties of GBFs [6]. Hence, it is essential to propose a new strategy to improve the mechanical properties of the GBFs incorporated with polyphenols. 

Microfibrillated cellulose (MFC), as a kind of renewable product, is well-known for its high elastic modulus and tensile strength as a mechanical reinforcement phase [24]. MFC is a form of nanocellulose in which the outer layer of the fibers has been shucked off via mechanical shearing, exposing the fibril bundles. The fibrils in MFC are much smaller in diameter compared to the original fibers and can form a network or a web-like structure [25]. More importantly, the microfibrils in MFC have a diameter in nanometers and length in micrometers, which makes them long and thin. This high aspect ratio makes the high-strength MFC useful in applications such as reinforcement of composites, films, and barriers. For instance, MFC can be incorporated into biopolymer-based films to improve their mechanical strength and ability to block water vapor permeation [24,26]. Besides, MFC is a renewable and biodegradable material, and the packaging industry is increasingly using renewable and biodegradable materials [27]. Therefore, these beneficial properties of the MFC fiber can help end-users in achieving lightweight and robust packaging solutions. MFC is supposed to be used to enhance the functions of GBFs. Herein, a facile strategy based on the principle of supramolecular chemistry was proposed for fabricating a strong and active GBF via the incorporations of gallic acid (GA) as an active crosslinking agent and MFC as a reinforcing agent. Specifically, the mechanical properties of the GBFs, as well as their structural features, microscopic morphology, optical properties, thermal stability, water vapor permeability, antioxidant properties, and crosslinking mechanism were determined. The supramolecular polymer network was established via forming multiple noncovalent interactions, thereby enhancing the mechanical strength of the resultant film. This strategy paves a new way toward producing high-performance and safe GBFs for active food packaging and preservation.

## 2. Materials and Methods

### 2.1. Materials

The gelatin (type B, limed-hide hydrolysis) used was purchased from Sinopharm Chemical Reagent Co., Ltd. (Shanghai, China). Its average molecular weight was about 100,000 g/mol. Glycerol (analytical grade) was provided by Sinopharm Chemical Reagent Co., Ltd. (Shanghai, China). Gallic acid was analytical grade and obtained from Tianjin Kemiou Chemical Reagent Co., Ltd. (Tianjin, China). Microfibrillated cellulose (1.8 wt%) was provided by China National Pulp and Paper Research Institute Co., Ltd. (Beijing, China), while 1,1-diphenyl-2-picrylhydrazyl (DPPH) was obtained from TCI Chemicals (Shanghai, China) and 2,2′-azinobis-3-ethylbenzthiazoline-6-sulfonate (ABTS) was obtained from Biotopped Life Science (Beijing, China). Methanol was analytical grade and provided by Macklin Biochemical Technology Co., Ltd. (Shanghai, China).

### 2.2. Preparation of Gelatin-Based Films

Gelatin-based films (GBFs) were prepared via a solution casting method [7,28]. Firstly, 10.0 g of gelatin, 4.0 mL of glycerol (40 *w*/*v*% of gelatin), and 0.5 g gallic acid (GA, 5.0 wt% based on gelatin) were dissolved in 96 mL distilled water. After continuous stirring at 60 °C for 30 min, microfibrillated cellulose (MFC, 0.5 wt%, 1.0 wt%, 3.0 wt% and 5.0 wt% of gelatin, based on the dry basis) was added to the gelatin solution dropwise and the solution pH was adjusted to around 4.0 using formic acid. After that, the solution was heated at 60 °C for 60 min with intense stirring. Besides, a pure gelatin film solution was prepared following the same method with no additions of gallic acid and MFC for comparison. Another gelatin film solution was prepared using a similar procedure with no introduction of MFC for clarifying the role of MFC. All the as-prepared solutions were ultrasonically degassed and then cast on leveled Teflon plates (14 cm × 14 cm) and dried at room temperature for 72 h. After peeling off from the plates, the dried films were air-conditioned at 25 °C and 50% RH for 48 h for further analyses. These film samples were designated as GF (neat gelatin film), GGF (gelatin and GA composite film), GGCF-1, GGCF-2, GGCF-3, and GGCF-4 (gelatin and GA mixed with MFC at 0.5 wt%, 1.0 wt%, 3.0 wt% and 5.0 wt%, respectively).

### 2.3. Structure Characterizations

The GBFs were analyzed using a Fourier-transform infrared spectrometer equipped with an attenuated total reflectance accessory (TENSOR 27, Brook, Germany). Each infrared spectrum (wavenumber, 4000~600 600~4000 cm^−1^) was collected at a resolution of 4 cm^−1^. The X-ray diffraction (XRD) patterns of the GBFs were obtained using an XRD diffractometer (X-D6, Persee, Beijing, China) in the diffraction angles of 2θ = 5~85° with a scanning speed of 0.4°/min using Cu Kα radiation (λ = 1.54 nm) with a nickel monochromator operating at the voltage of 36 kV and the current of 20 mA. The thermal stability of each GBF was evaluated using a thermogravimetric analyzer (TGA/DSC 3+, Mettler Toledo, Zurich, Switzerland). For this, about 10 mg of film sample was heated from 30 to 800 °C at the heating rate of 10 °C/min under the N2 atmosphere. The surface and cross-sections of the GBFs were investigated using a field emission scanning electron microscope (FESEM, Apreo 2C SEM, Thermo Scientific, Waltham, MA, USA) in the high-vacuum mode with the acceleration voltage of 5 kV. The surface roughness of the film samples was observed using an atomic force microscope (AFM, AFM5100W, Hitachi, Tokyo, Japan) [29].

### 2.4. Optical Properties Analyses

The surface color of the film samples was measured using a color measurement instrument (SC-80C, Jingyi Kangguang, Beijing, China) with the standard white color plate (L = 95.41, a = −0.46, b = 2.01) as the background. The illuminant used was a D65 standard light source, and the field of view was 10°. The total color difference (ΔE) was calculated according to Equation (1):

(1) $$∆E=\sqrt{∆L^{2}+∆a^{2}+∆b^{2}}$$

where ΔL, Δa, and Δb are the difference between each color value of the film sample and the standard plate, respectively [30].

The UV–vis spectra of GBFs were recorded using a UV–vis spectrophotometer (TU-1810, Persee, Beijing, China). In particular, the optical properties of the films, including the UV barrier property and transparency, were assessed by recording light transmittance percentages at 280 nm (T_280_) and 660 nm (T_660_), respectively. Moreover, the appearances of different films were recorded using a digital camera [31].

### 2.5. Determination of Physical Properties 

Water vapor permeability (WVP) was measured using a gravimetric method according to the method described by Riahi et al. [31] and Yu et al. [22] with a slight modification. The film samples (the diameter was 50 mm) were mounted on top of a glass jar (depth = 95 mm; inner diameter = 30 mm) containing 50 mL of distilled water and sealed in such a way that the evaporation of water from the cup took place only through the sample film. The assembled glass jars were weighed and stored in a desiccator controlled at 25 °C and 25% RH. The weight of the jar was measured at 0, 1, 2, 4, 6, 9, 12 h at 25 °C, and the weight loss of the jar versus time was plotted to measure the water vapor transmission rate (WVTR, g/m^2^ s) from the slope of the plot. The WVP (g·m/m^2^·Pa·s) of the film was calculated using Equation (2):

(2) $$\mathrm{WVP}=\frac{\mathrm{WVTR}\;\times \;L}{∆P}$$

where L represents the mean film thickness (m) and ΔP is the partial water vapor pressure difference (Pa) between the two sides of the film; ∆p was calculated according to the formula: ∆p = Ps × (R1 − R2), where Ps is the saturation vapor pressure of water at 25 °C (Pa), R1 is the relative humidity inside the jar (100%), and R2 is the relative humidity in the desiccator (25%). The surface water contact angle (WCA) of each GBF was measured with a contact angle meter (JC2000DM, Beijing Zhongyi Kesin Technology Co., Ltd., Beijing, China). Ten microliters of deionized water were used to test the surface contact angle of the GBFs. Dissolution of the GBFs in distilled water was simply evaluated by adding a piece of film (0.5 cm × 3.0 cm) into a reagent bottle containing 10 mL of distilled water. After swelling for 12 h, the appearance of the film was recorded using a digital camera before/after shaking well.

The thickness of the GBFs was measured at three random points using a digital micrometer with an accuracy of 1 μm, and the average value was used. The tensile strength (TS) and percentage elongation at break (EB) of the GBFs (25 mm × 150 mm) were analyzed following the ASTM D 882-88 standard method using a universal testing machine (AI-7000SN, Gotech, Dongguan, China). The apparatus ran in the tensile mode, with the initial grip separation of 15 mm and the cross-head speed of 500 mm/min. 

### 2.6. Antioxidant Activity Evaluation

The antioxidant capacity of the GBFs was evaluated with a UV–vis spectrophotometer (TU-1810, Persee, Beijing, China) according to the ABTS radical and DPPH radical scavenging methods described by Roy et al. [1] with a slight modification. A GBF in the amount of 0.1 g was placed in a tube filled with 5.0 mL of methanol solution (70%, *v*/*v*) to extract the active compounds. After incubation at 25 °C for 24 h, the supernatant was collected to determine the radical scavenging activities of ABTS and DPPH.

For the ABTS radical scavenging activity assay, 7.00 mM of the aqueous ABTS solution and 2.45 mM of the K2S2O8 solution were blended with a 1:1 volume ratio and stood for 16 h in the dark. The as-prepared ABTS radical solution was then diluted with methanol until the absorbance value at 734 nm was about 0.70 ± 0.02. After that, 1.0 mL of the GBF extract solution and 3.0 mL of this diluted solution were blended thoroughly and incubated in the dark at 25 °C for 10 min before testing absorption at 734 nm. The ABTS radical scavenging activity (%) was calculated according to Equation (3):

(3) $$\mathrm{Scavenging}\;\mathrm{activity}/\%=\frac{A_{0}-A_{T}}{A_{0}}\times 100$$

where A_0_ and A_T_ represent the absorbance of the ABTS solution of the control and the GBF sample, respectively.

For the DPPH radical scavenging activity measurement, 4 mg of DPPH were dissolved in 100 mL of methanol to prepare the DPPH methanolic solution. After that, 4.0 mL of this solution and 2.0 mL of the GBF extract solution were mixed well and stood in the dark for 30 min, and then the absorbance at 517 nm was measured. As a control, the DPPH solution was prepared without adding the film solution, which was replaced with the methanol solution. The radical scavenging activity (%) of DPPH was calculated according to Equation (4):

(4) $$\mathrm{Scavenging}\;\mathrm{activity}/\%=\frac{A_{c}-A_{s}}{A_{c}}\times 100$$

where A_c_ and A_s_ represent the absorbance for the control and the GBF sample solution, respectively.

### 2.7. Statistical Analysis

The experimental data were shown as the means ± standard deviations from at least three parallel experiments. The data were subjected to the one-way ANOVA analysis followed by Tukey’s post hoc test for statistical analysis. The mean values were statistically significant if the probability values were less than 0.05 (*p* < 0.05).

## 3. Results and Discussion

### 3.1. Structure Features of MFC

**Figure 1:**
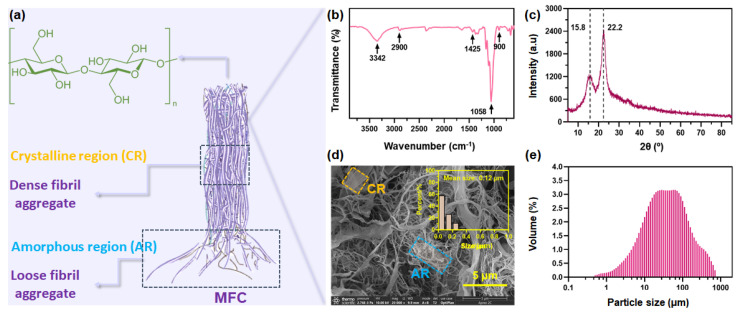
Schematic illustration (**a**) with permission from Springer 2019 [34], FTIR spectrum (**b**), XRD spectrum (**c**), SEM image (**d**) and particle size (**e**) of microfibrillated cellulose (MFC).

As mentioned above, microfibrillated cellulose (MFC) is a kind of cellulose in which the outer layer of the fibers has been shucked off using mechanical shearing, exposing the fibril bundles with amorphous and crystalline domains (Figure 1a). The structure features of MFC were first clarified using FTIR, XRD, SEM, and particle size measurement. As shown in Figure 1b, the pristine MFC presents some typical peaks for cellulose: hydrogen-bonded –OH stretching at 3342 cm^−1^, –CH stretching at 2900 cm^−1^, –CH_2_ bending at 1425 cm^−1^, –C–O stretching at 1058 cm^−1^, and –CH bending or –CH_2_ stretching at 900 cm^−1^. These typical peaks indicate the amorphous structure [32]. Figure 1c illustrates the X-ray diffraction pattern for MFC powder, which is a typical diffractogram reported for semi-crystalline polymeric materials. The overlapping peaks positioned at diffraction angles 2θ of ~15.8° corresponding to the amorphous region and another diffraction peak positioned at a diffraction angle 2θ of ~22.2° corresponding to the crystalline region were observed [20].

To clarify natural aggregates of cellulose nano- and microfibrils in the MFC, SEM observation of powdery MFC was conducted. Figure 1d illustrates the representative SEM micrographs of the MFC used in this work. Low-aspect-ratio fiber aggerates and high-aspect-ratio fibril aggregates could be observed. The average diameter of MFC was calculated to be 0.12 μm. This confirmed the structural feature of MFC, ensuring the potential functionalities that can be offered by MFC in the reinforcement of GBF. MFC has a high affinity to itself and the materials with many hydroxyl groups, especially water. Owing to the dominance of hydroxyl groups, polymeric MFC is very reactive with water [33]. As a result, in an aqueous MFC solution, nano- and microfibrils might tend to aggregate into fiber bundles in larger sizes via both intra- and intermolecular hydrogen bonds, which can be verified by the larger particle size of MFC in water shown in Figure 1e. This feature may endow MFC with a dose–effect relationship between its additive amount and the reinforcing effect when it is used as a reinforcer in the fabrication of GBF.

### 3.2. FTIR and XRD Analyses of GBFs

**Figure 2:**
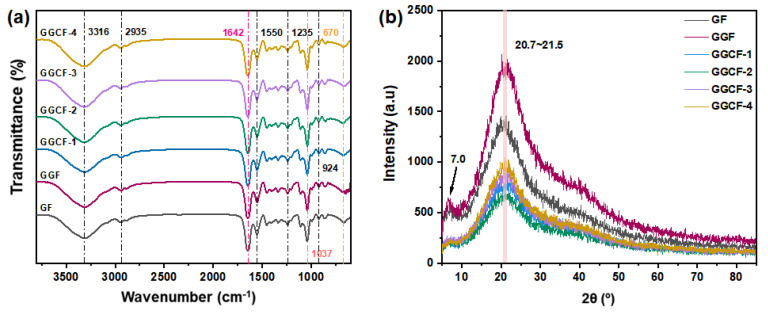
FTIR (**a**) and XRD (**b**) spectra of gelatin-based films (GBFs).

The FTIR spectra of the GBFs are shown in Figure 2a. The pure gelatin film (GF) had characteristic peaks at 3316 cm^−1^ and 2935 cm^−1^. They are respectively assigned to the O–H and C–H stretching vibrations in the gelatin structure [35]. At 1642 cm^−1^, there was a peak ascribed to the amide-I vibration of the C=O group connected to COO^–^. For the peak at 1550 cm^−1^, it was assigned to the amide-II vibration because of the N–H bending and C–N stretching. Furthermore, an amide-III vibration band appeared at 1235 cm^−1^, suggesting the vibrations of C–N and N–H groups of bound amides in the plane [36]. For GF, the amide-I, amide-II, and amide-III vibration bands’ positions were similar to those of gelatin reported in other works. Moreover, the spectra showed the amide-V band at 670 cm^−1^, which may be the feature of low-molecular-weight peptides in gelatin [31]. The peak at 1037 cm^−1^ was due to the in-plane bending vibration of the –OH group of glycerol [37]. Notably, it can be seen that the characteristic absorption peaks of GA and MFC overlapped in the spectra of gelatin and glycerol. Thus, the spectra of the GF, GGF, and GGCFs are highly identical. When the GA and MFC were added, there was no significant difference observed in the band position, indicating that no new chemical bonding formed in the GBFs. 

The change in the crystallinity of the GBFs with the addition of GA and MFC was evaluated using XDR. Figure 2b shows the XRD patterns of the different GBFs. The neat gelatin film (GF) without GA and MFC exhibited a representative amorphous structure with a broad peak in the 2θ range of 10.0~25.0° [38]. The XRD pattern of the GGCFs did not present a distinct characteristic peak of GF and GGF with the introduction of MFC, which might be ascribed to the overlapping effect of the gelatin matrix on MFC (diffraction angle 2θ of ~15.8° and ~22.2°) with low concentrations (0.5 wt% to 5.0 wt%). Furthermore, the integrated intensity of the diffraction peak with an angle 2θ of ~8° for GGCFs was lower than that for GGF and GF, suggesting that a decreased amount of triple-helix configuration was present within the GGCF matrix compared to the GF and GGF matrix [29]. This decreased formation of triple-helix configurations might be attributed to the higher molecular weight of the GGCF system compared to GF and GGF systems, which might restrict molecular mobility during film formation [29]. This also laterally indicated that introducing MFC might improve the stability of the GF’s network structure.

### 3.3. Microstructure of GBFs

**Figure 3:**
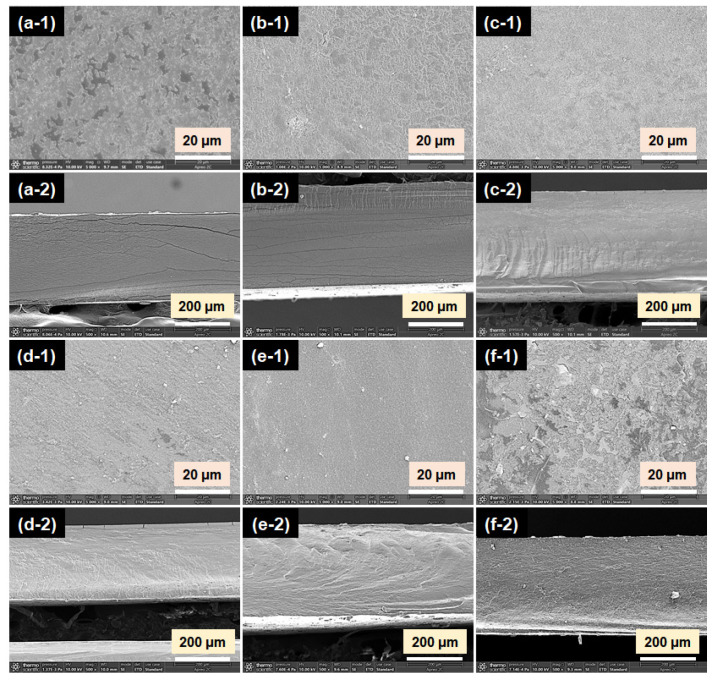
SEM micrographs of surfaces (**1**) and cross-sections (**2**) of GBFs: GF (**a**); GGF (**b**); GGCF-1 (**c**); GGCF-2 (**d**); GGCF-3 (**e**); GGCF-4 (**f**).

The SEM images of the surface and cross-sections of the GBFs are shown in Figure 3. It can be observed that some pores existed on the surface of GF, and the pores still existed when GA was introduced. With the addition of MFC, the number of pores gradually decreased, and the pores were hard to observe when the concentration of MFC was not higher than 3.0 wt% of gelatin. When 5.0 wt% of MFC was incorporated in the GBF, there were a lot of pores on the surfaces of GBFs (GGCF-4), which might be due to the aggregation of excess MFC. Meanwhile, the film structure became rather tanglesome and discontinuous, which might harm the mechanical properties of the film. As presented in the cross-sectional micrographs (Figure 3(a-2)–(f-2)), there were many cracks in GF and GGF. With the introduction of MFC, the cracks gradually disappeared in the cross-sections. This suggested that MFC performed an obvious filling effect on the GBFs, resulting in the more compact structure of GBFs. This might lead to the increase in tensile strength of GBFs.

**Figure 4:**
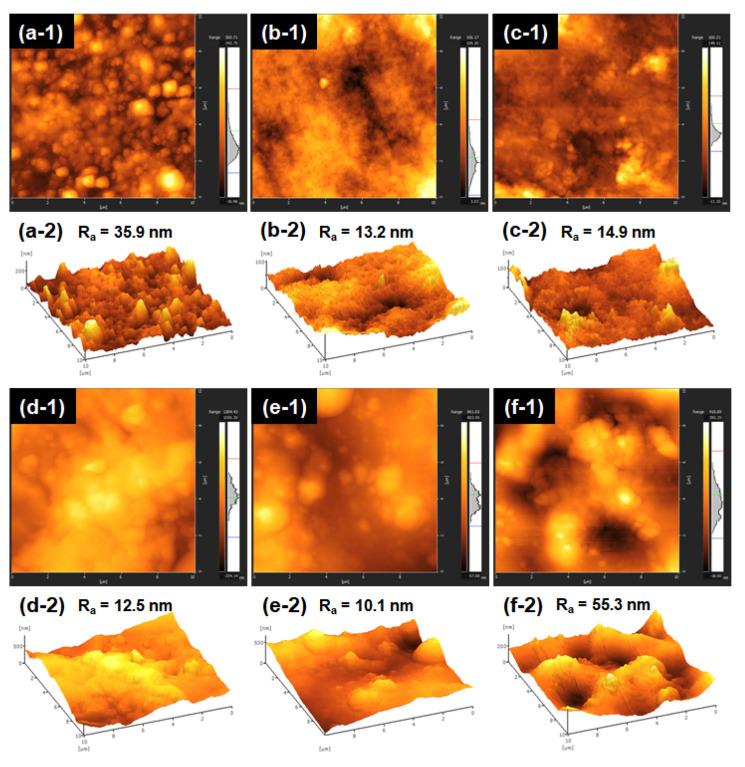
AFM micrographs of surfaces of GBFs with a size of 10 µm × 10 µm: GF (**a**); GGF (**b**); GGCF-1 (**c**); GGCF-2 (**d**); GGCF-3 (**e**); GGCF-4 (**f**).

Furthermore, AFM observation of the GBF was conducted to assess its surface roughness (Figure 4). The measured roughness value (Ra) of the GBF matched the SEM observations. A higher Ra value means higher roughness [39]. For GF, the Ra value was up to 35.9 nm, indicating the high roughness of the film. The surface roughness of GBFs increased first as the concentration of MFC increased and then decreased as the MFC addition was up to 5.0 wt% of gelatin. The Ra was highest for 5.0 wt% because of the aggregation of MFC in the polymer matrix. Similarly, an aggregation effect of cellulose nanofibrils (CNF) at a high concentration had also been observed in gelatin films [40] and starch nanocomposites [41]. In light of the above results and as inspired by the XRD results, excessive MFC would also have the possibility to decrease the molecular mobility of the film’s components, thereby leading to a reduction in the elongation at break of the GGCFs.

### 3.4. Surface Color and Optical Properties

**Figure 5:**
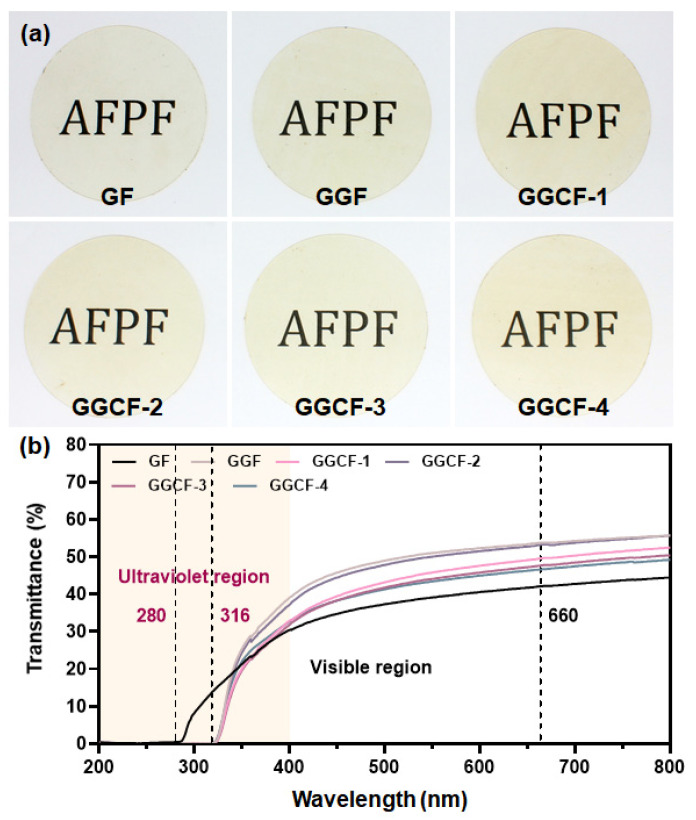
Appearance (**a**) and UV–vis spectra (**b**) of GBFs; AFPF: active food packaging film.

The physical appearance of the packaging film is a key parameter, especially the color, which usually dominates the visibility of the packaged product and consumer’s acceptance [12]. With the color of the GBFs shown in Table 1, the L* value displays an obvious decrease (*p* < 0.05), whereas the b* and ΔE values significantly increased (*p* < 0.05) with the addition of GA in the films. After incorporating MFC, the L* value further decreased while the b* value increased and the a* value had no significant change. With more MFC incorporated, the L*, a*, b* did not change significantly overall, but the ΔE value generally increased. The results indicated that introducing MFC made the GBFs yellower, which can be visually observed from the appearances of GBFs shown in Figure 5a. In short, GA had a significant effect on the color of the GBF, resulting in a darker and yellower color. As was reported by Riahi et al., the L* and b* values of protein-based films would be markedly increased after the introduction of plant extracts [31], and the ΔE value of the films would also be greatly increased, which was in accord with our results. 

Next, the light transmittance of GBFs was recorded in the region of 200–800 nm, aiming to investigate the UV barrier and transparency properties. Figure 5b shows the UV–vis spectra of different GBFs. The polypeptide chains of gelatin contain aromatic amino acids (tyrosine and phenylalanine) that can absorb UV radiation [42]. At 280 nm, all the GBFs presented a negligible transmittance value (Table 1). With the addition of GA, the resistance to UV light transmission of GBFs in the UV region 280–316 nm was further improved due to the aromatic ring structure with anti-ultraviolet properties [6]. The addition of MFC showed no negative impact on the UV light absorption function of GBFs. In the visible region of 400–800 nm, the GF showed the lowest transmission of light while GGF exhibited the highest transmission of light. With the incorporation of MFC, the visible light transmittance of GBFs had a slight decrease (Table 1). This might be due to the difference in the dispersion and aggregation of MFC in different GBFs. In short, the addition of GA could considerably improve the UV resistance and visible light transmittance of the GBFs. Meanwhile, the introduction of MFC would not cause obvious damage to the above properties.

### 3.5. WVP and WCA of GBFs

As is well-known, moisture migration is an important factor that can affect the shelf life of food products [43]; thus, WVP of the GBFs was further measured. Compared with GF, the addition of GA resulted in an increment in the WVP of the GBFs (Table 2). However, there was an obvious decrease in WVP for the GBFs (GGCFs) incorporated with MFC. When 5.0 wt% MFC was incorporated, WVP of a GBF (GGCF-4) was much lower than that of GF. This indicated that the capability to block water vapor permeation was improved. Similarly, Ortiz et al. also reported that the GBFs had lower WVP after the introduction of MFC. This effect could be attributed to a successful dispersion of nanofibers within the protein matrix that helped to construct a more tortuous path for the passage of water molecules through the film [44]. Meanwhile, the introduced MFC might provide more intermolecular crosslinking in the film matrix via forming hydrogen bonding and hydrophobic interactions [23]. This feature would show unpleasant impacts on some hydrophilic domains in the film matrix, thereby leading to the declined hydrophilicity of the films [22]. 

Table 2 shows that the WCA of the GF was 69.30°, which was increased to 75.16° through adding GA. When only 0.50 wt% MFC was added to a GBF, the WCA further increased to 76.35°. As the concentration of MFC further increased, the WCA was also continuously increased, which could be up to 80.22° when 5.0 wt% MFC was introduced. This suggested that the introduction of MFC could lead to an increase in hydrophobicity of the GBFs, which was beneficial for improving the water vapor barrier property of GBFs to potentially protect the packaged products from putrefaction. Figure S1 also shows that the as-prepared GBFs could be completely dissolved in distilled water after swelling for 12 h, which suggests the easy treatability of these GBFs.

### 3.6. Mechanical Strength

**Figure 6:**
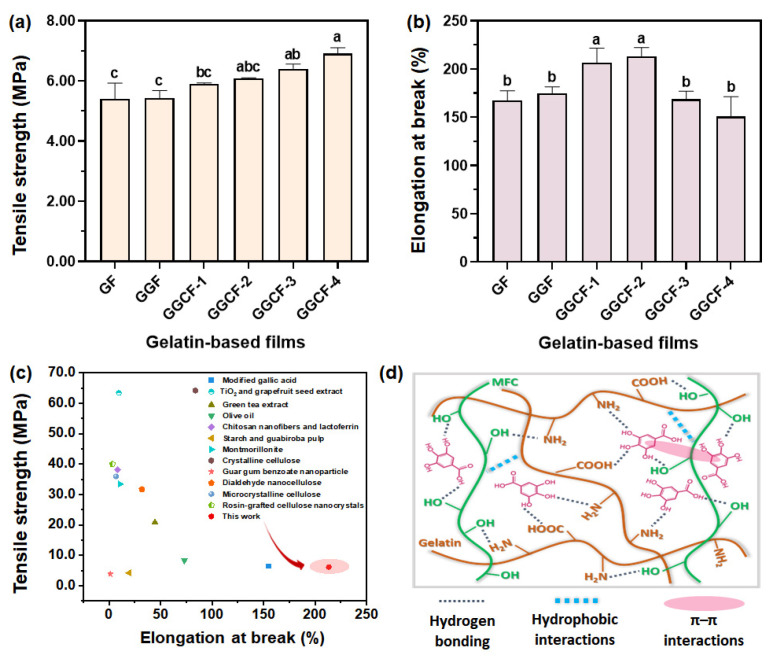
Tensile strength (**a**) and elongation at break (**b**) of the GBFs; (**c**) comparison of the mechanical strength of different GBFs; (**d**) the proposed reinforcing mechanism of MFC on the gelatin-based film. The results are expressed as the means ± SD (n = 3), the columns marked with different lowercase letters are significantly different from each other at *p* < 0.05.

As was previously reported, the interactions of gelatin chains with other components, e.g., antioxidant substances, plasticizers, water, and reinforcing agents can markedly impact the mechanical properties of the resultant composite films [45]. Besides, the component content of these components can also play an important role in the mechanical properties of the resultant composite films. Herein, the mechanical properties of all the GBFs were determined, including tensile strength (TS) and elongation at break (EB). It can be seen in Figure 6 that the TS and EB of the GBF had no obvious change when the GA was added. After the introduction of MFC (0.5 wt% to 5.0 wt%), there was a significant increase in the TS of GGCFs while the EB of GGCFs (MFC-added films) increased first and then decreased. In particular, when the MFC concentration was set to be 0.50 wt% and 1.0 wt%, the EB of the GBFs was higher than that of GF and GGF and could be up to about 200%, which was much higher than that of the GBFs reinforced by other additives (Figure 6c and Table S1). After increasing the MFC concentration, the EB of GBFs decreased to be comparable with that of GF and GGF. 

Overall, the introduction of MFC significantly improved the mechanical properties of the GBF incorporated with GA. This favorable combination of high TS and EB was mainly ascribed to the tough noncovalent interactions within the GGCF. The crystalline MFC molecules could result in the formation of rigid crosslinking domains in the gelatin matrix. The amorphous chains of MFC could just act as a soft constituent to keep the flexibility of the gelatin films. Besides, the hydrophilic MFC weakened the interaction between proteins, thereby reducing cohesion and increasing the plasticity of the blend matrix, so that the resulting GGCF had higher flexibility [46]. 

During the stretching process, the energy could be effectively dissipated by the synergistically improved dynamic hydrogen bonds, hydrophobic interactions and π–π interactions in the GGCF (Figure 6d) [47] that could act as sacrificial bonds [48]. These multiple interfacial interactions aided the formation of a strengthened supramolecular network in these composites to update the adhesion and cohesion of the GGCF. In this work, the tensile strength of the GBFs was updated with the incorporation of MFC. However, in comparison with the traditional plastic materials, the GGCFs have no sufficiently strong mechanical properties, which needs to be ameliorated in the future, especially the TS.

### 3.7. Thermal Stability

**Figure 7:**
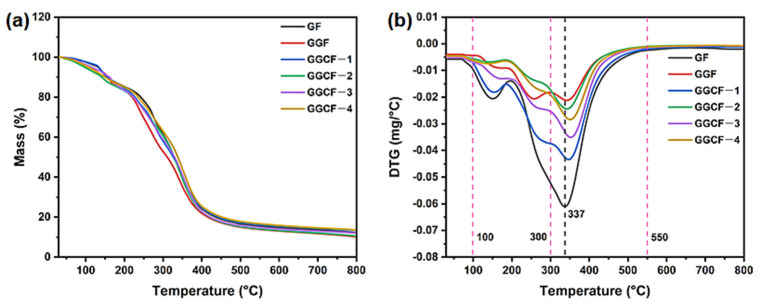
TG (**a**) and DTG curves (**b**) of GF, GGF, and different GGCFs.

The thermal stability of a GBF is very important for its practical application in complex environments. Figure 7 illustrates the thermal stability of various GBFs investigated using the TG–DSC measurement. There are three degradation stages for the thermal decomposition of GF. The first stage occurs below 100 °C and is mainly composed of weight loss due to the desorption of absorbed water [49]. In the second stage (from 100 °C to 300 °C), there is a weight loss of 36% caused by the degradation of glycerol in the GBF [50]. In the final stage (from 300 °C to 550 °C), peptide bonds and sidechain groups in amino acid residues are degraded. With a further increase in temperature, the gelatin matrix began to carbonize, which resulted in weight loss during the decomposition process [47]. 

The incorporation of GA and MFC led to the peak temperature (T_max_) of the GBF increasing from 337 °C (GF) to 355 °C (GGCF-3). This increment was associated with the formation of a compact char layer in the GBF matrix due to the presence of phenolic hydroxyl groups and aromatic char produced by GA at high temperatures. When the GA and MFC were heated to high temperatures, more carbon residue and char would be formed, causing a loss in the combustion heat of GBFs [51]. During the heating process, the GA and MFC contributed to the formation of the char layer, hindering the mass transfer and heat flow between the condensed phase and the combustible gas, thereby accelerating the dehydration and carbonization of the gelatin matrix [52]. Therefore, the GA and MFC prevented further erosion of the underlying gelatin matrix from heat flux caused by thermal decomposition, resulting in the improved thermal stability of GBFs [47]. The updated thermal stability of GBFs provides a promising prospect for the application of gelatin-based packaging materials in harsh environments.

### 3.8. Antioxidant Activity

**Figure 8:**
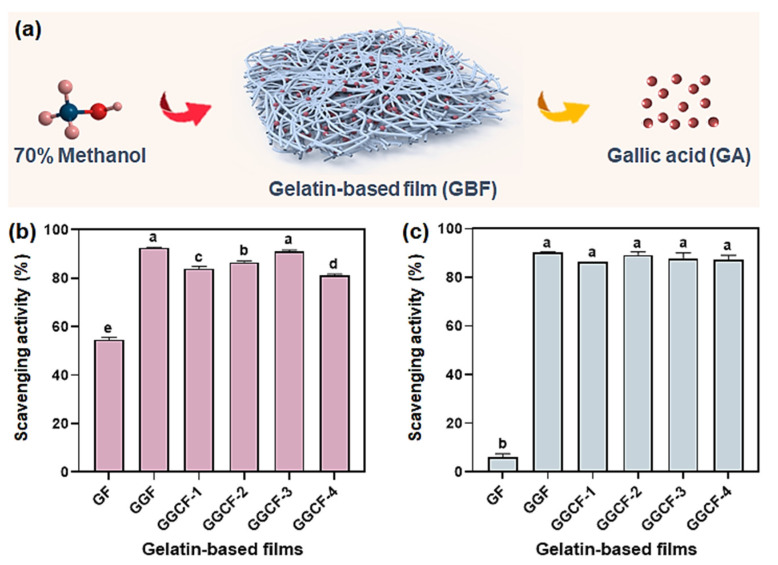
Schematic diagram of the release of GA from a GBF in an aqueous methanol solution (**a**); ABTS (**b**) and DPPH (**c**) radical scavenging capacities of GBFs. The results are expressed as the means ± SD (n = 3), the columns marked with various lowercase letters are significantly different from each other at *p* < 0.05.

The antioxidant ability of GBFs was evaluated using ABTS and DPPH free radical scavenging tests. Figure 8a illustrates that the active compounds, particularly GA, should be released from the network structure of GBF in a methanol aqueous medium. Figure 8a illustrates that the GF performed moderate ABTS free radical scavenging activity while it showed weak DPPH free radical scavenging property (Figure 8b). GA was previously proven to possess remarkable antioxidant activities, including free radical scavenging capacity [20]. Thus, the scavenging effects of GBFs on the two kinds of free radicals were significantly improved with the introduction of GA. Moreover, a notable concentration-dependent change in ABTS free radical scavenging activity was observed when MFC (1.0~5.0 wt% of gelatin) was integrated into the films. Gelatin is a well-known protein source for antioxidant hydrolysates [53] due to the presence of phenolic amino acids. According to the work reported by Guo et al., the neat gelatin film had weak DPPH free radical scavenging capacity [6], which was similar to the present DPPH free radical scavenging activity of GF. Moreover, it can be observed that the introduction of MFC showed no impact on the DPPH free radical scavenging capacity of GBFs. In comparison, the DPPH radical scavenging activity of polypropylene (PP)-based composite films was 73% when the BHT concentration was 3% of the composite film [54]. Besides, the DPPH radical scavenging activity of fish skin gelatin film incorporated with BHT at the concentration of 200 ppm was not higher than 10% [55].

In summary, the abovementioned PP-based composite films had higher TS (up to 69 MPa) and EB (up to 704%) than the as-prepared GBFs [54]. Some of the GBFs reinforced with other additives might have had higher TS (up to 64), but their EBs were generally much lower than those of the as-prepared GBFs (Table S1). Overall, in light of the aforementioned results, it was suggested that the antioxidant ability of GBFs incorporated with GA was favorable, and the introduction of MFC would not cause obvious negative effects and could improve the mechanical strength of GBFs when a suitable MFC concentration was employed, which is beneficial for the fabrication of active and robust GBFs. As-prepared GBFs show potential to be used as active edible food films or other active food packaging materials for food preservation.

## 4. Conclusions

In this work, a robust and active gelatin-based film (GBF) with improved UV-blocking ability, water vapor barrier and antioxidant activity was successfully fabricated. The interconnected crosslinking network was established in the gelatin matrix by integration with gallic acid (GA) and microfibrillated cellulose (MFC) via forming multiple noncovalent interactions between gelatin, GA, and MFC chains, including hydrogen bonds, hydrophobic interactions, and π–π interactions. These interactions could act as dynamic sacrificial bonds to promote effective energy dissipation during stretching so that the resultant GBF had higher mechanical properties than a pure gelatin film (GF). With introducing MFC at a concentration of 1.0 wt%, MFC was evenly dispersed in the gelatin matrix to endow the film with low surface roughness and compact structure, resulting in corresponding improvements of 12.8% for tensile strength and 27.6% for elongation at break relative to the neat gelatin film (GF). The UV-blocking performance, water vapor barrier, thermal stability, and antioxidant activity of GBFs were also significantly improved relative to the GF after introducing the aromatic structure of GA and nano-/microfibrils in MFC. This work paves a promising way toward the facile preparation of multifunctional GBFs that have great potential to be used in fabricating active and safe food packaging materials.

## Figures and Tables

**Table 1:** Apparent color and light transmittance of GBFs.

Films	L ^*^	A ^*^	B ^*^	∆E	T_280_/%	T_660_/%
GF	89.90 ± 0.44 ^a^	−0.84 ± 0.31 ^a^	8.29 ± 0.58 ^c^	8.30	0.4	42.0
GGF	88.16 ±0.44 ^b^	−0.69 ± 0.43 ^a^	10.77 ± 0.20 ^b^	11.25	0.3	53.0
GGCF-1	87.86 ± 0.16 ^b^	−0.63 ± 0.32 ^a^	12.08 ± 0.52 ^ab^	12.47	0.3	49.4
GGCF-2	87.30 ± 0.29 ^bc^	−0.30 ± 0.58 ^a^	12.30 ± 0.88 ^ab^	12.98	0.3	53.1
GGCF-3	86.78 ± 0.02 ^c^	−0.90 ± 0.34 ^a^	12.71 ± 0.13 ^a^	13.62	0.3	47.5
GGCF-4	87.41 ± 0.31 ^bc^	−0.72 ± 0.24 ^a^	12.78 ± 0.32 ^a^	13.30	0.3	46.5

* Data are expressed as the means ± SD (n = 3), and the different lowercase superscripts in the same column indicate significant differences (*p* < 0.05).

**Table 2:** Water vapor permeability (WVP) and water contact angle (WCA) of GBFs.

Films	WVP (×10^−8^ g·m^−1^·Pa^−1^·s^−1^) *	WCA (deg.) *
GF	3.985 ± 0.001 ^abc^	69.30 ± 1.41 ^b^
GGF	4.379 ± 0.225 ^ab^	75.16 ± 0.49 ^a^
GGCF-1	3.768 ± 0.194 ^bc^	76.35 ± 0.81 ^a^
GGCF-2	3.894 ± 0.082 ^bc^	79.25 ± 1.61 ^a^
GGCF-3	4.093 ± 0.079 ^ab^	79.48 ± 2.21 ^a^
GGCF-4	3.634 ± 0.189 ^c^	80.22 ± 2.27 ^a^

* Data are expressed as the means ± SD (n = 3), and the different lowercase superscripts in the same column indicate significant differences (*p* < 0.05).

## Data Availability

The data presented in this study are available on request from the corresponding author.

## Supplementary Materials

The following are available online at https://www.mdpi.com/article/10.3390/foods10112831/s1, Table S1: Comparison of the reinforcing effect of different additives for the gelatin-based film (GBF). Figure S1: Dissolution of GBFs in distilled water.
