# Supplementary material for: Active and Robust Composite Films Based on Gelatin and Gallic Acid Integrated with Microfibrillated Cellulose
Source: Foods. 2021 Nov 17;10(11):2831. doi: 10.3390/foods10112831 (PMC8619323; doi:10.3390/foods10112831)
Supplement: Supplementary file 1 [file foods-10-02831-s001.zip › foods-1435772-supplementary.pdf]

Supplementary Material

Active and Robust Composite Films Based on Gelatin and Gallic Acid Integrated with Microfibrillated Cellulose

Yinghua Luo [1], Yanbei Wu [2,*], Yali Wang [2], Liangli (Lucy) Yu [3]

[1] College of Food Science and Nutritional Engineering, National Engineering Research Centre for Fruits and Vegetables Processing, Key Laboratory of Storage and Processing of Fruits and Vegetables, Ministry of Agriculture, Engineering Research Centre for Fruits and Vegetables Processing, Ministry of Education, China Agricultural University, Beijing 100083, China

[2] School of Food and Health, Beijing Technology and Business University, Beijing 100048, China

[3] Department of Nutrition and Food Science, University of Maryland, College Park, MD 20742, USA

Corresponding author

Yanbei Wu, E-mail: yanbeiwu@btbu.edu.cn.

Table S1. Comparison of the reinforcing effect of different additives for gelatin-based film (GBF).

Additive	TS/Mpa	EB/%	References
Modified gallic acid	6.4	155.1	[1]
TiO_2 and grapefruit seed extract	63.4	9.6	[2]
Green tea extract	20.8	44.6	[3]
Olive oil	8.3	73.2	[4]
Chitosan nanofibers and lactoferrin	38.1	8.3	[5]
Starch and *guabiroba* pulp	4.2	19.7	[6]
Montmorillonite	33.4	10.6	[7]
Crystalline cellulose	64.2	83.7	[8]
Guar gum benzoate nanoparticle	3.8	1.3	[9]
Dialdehyde nanocellulose	31.7	32.0	[10]
Microcrystalline Cellulose	35.9	6.8	[11]
Rosin-grafted cellulose nanocrystals	40.0	3.3	[12]
This work	6.1	213.4	-

Water solubility of GBFs

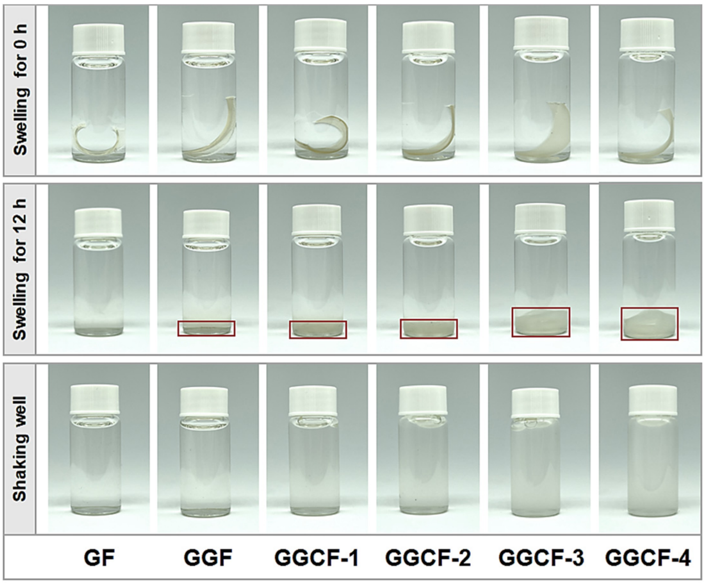

Figure S1. Dissolution of GBFs in distilled water.

References

1. Guo, L.; Qiang, T.; Ma, Y.; Ren, L.; Zhu, C. Biodegradable Anti-Ultraviolet Film from Modified Gallic Acid Cross-linked Gelatin. *ACS Sustain. Chem. Eng.* **2021**, *9*, 8393–8401.

2. Riahi, Z.; Priyadarshi, R.; Rhim, J.; Bagheri, R. Gelatin-based functional films integrated with grapefruit seed extract and TiO2 for active food packaging applications. *Food Hydrocoll.* **2021**, *112*, 106314.

3. Li, J.; Miao, J.; Wu, J.; Chen, S.; Zhang, Q. Preparation and characterization of active gelatin-based films incorporated with natural antioxidants. *Food Hydrocoll.* **2014**, *37*, 166–173.

4. Ma, W.; Tang, C.; Yin, S.; Yang, X.; Wang, Q.; Liu, F.; Wei, Z. Characterization of gelatin-based edible fi lms incorporated with olive oil. *Food Res. Int.* **2012**, *49*, 572–579.

5. Tavassoli, M.; Sani, M.A.; Khezerlou, A.; Ehsani, A.; McClements, D.J. Multifunctional nanocomposite active packaging materials: Immobilization of quercetin, lactoferrin, and chitosan nanofiber particles in gelatin films. *Food Hydrocoll.* **2021**, *118*, 106747.

6. Malherbi, N.M.; Schmitz, A.C.; Grando, R.C.; Bilck, A.P.; Yamashita, F.; Tormen, L.; Fakhouri, F.M.; Velasco, J.I.; Bertan, L.C. Corn starch and gelatin-based films added with guabiroba pulp for application in food packaging. *Food Packag. Shelf Life* **2019**, *19*, 140–146.

7. Ribeiro, H.L.; Brito, E.S.; Azeredo, H.M.C. Montmorillonite as a reinforcement and

color stabilizer of gelatin films containing acerola juice. *Appl. Clay Sci.* **2018**, *165*, 1–7.

8. Bhowmik, S.; Islam, J.M.M.; Debnath, T.; Miah, M.Y.; Bhattacharjee, S.; Khan, M.A. Reinforcement of gelatin-based nanofilled polymer biocomposite by crystalline cellulose from cotton for advanced wound dressing applications. *Polymers (Basel).* **2017**, *9*, 222.
9. Kundu, S.; Das, A.; Basu, A.; Abdullah, F.; Mukherjee, A. Guar gum benzoate nanoparticle reinforced gelatin films for enhanced thermal insulation , mechanical and antimicrobial properties. *Carbohydr. Polym.* **2017**, *170*, 89–98.
10. Kwak, H.W.; Lee, H.; Park, S.; Lee, M.E.; Jin, H.J. Chemical and physical reinforcement of hydrophilic gelatin film with di-aldehyde nanocellulose. *Int. J. Biol. Macromol.* **2020**, *146*, 332–342.
11. Pan, L.; Li, P.; Tao, Y. Preparation and Properties of Microcrystalline Cellulose/Fish Gelatin Composite Film. *Materials (Basel).* **2020**, *13*, 4370.
12. Leite, L.S.F.; Bilatto, S.; Paschoalin, R.T.; Soares, A.C.; Moreira, F.K. V; Oliveira Jr, O.N.; Mattoso, L.H.C.; Bras, J. Eco-friendly gelatin films with rosin-grafted cellulose nanocrystals for antimicrobial packaging. *Int. J. Biol. Macromol.* **2020**, *165*, 2974–2983.
